# Leukocyte telomere length throughout the continuum of colorectal carcinogenesis

**DOI:** 10.18632/oncotarget.24431

**Published:** 2018-02-07

**Authors:** Cornelia Zöchmeister, Stefanie Brezina, Philipp Hofer, Andreas Baierl, Michael M. Bergmann, Thomas Bachleitner-Hofmann, Judith Karner-Hanusch, Anton Stift, Armin Gerger, Gernot Leeb, Karl Mach, Sivaramakrishna Rachakonda, Rajiv Kumar, Andrea Gsur

**Affiliations:** ^1^ Medical University Vienna, Department of Medicine I, Institute of Cancer Research, Vienna, Austria; ^2^ University of Vienna, Department of Statistics and Operations Research, Vienna, Austria; ^3^ Medical University Vienna, Department of Surgery, Vienna, Austria; ^4^ Medical University of Graz, Division of Oncology, Department of Internal Medicine, Graz, Austria; ^5^ Hospital Oberpullendorf, Burgenland, Austria; ^6^ German Cancer Research Center, Division of Molecular Genetic Epidemiology, Heidelberg, Germany

**Keywords:** telomere length, colorectal cancer, adenoma, cancer epidemiology

## Abstract

Considering the high prevalence of colorectal cancer (CRC) and relatively high mortality there is strong interest in identification of clinically relevant biomarkers. Telomere shortening is supposed to contribute to genomic instability and crucially involved in process of carcinogenesis. Peripheral blood leukocyte (PBL) telomere length was previously investigated in several studies as potential biomarker for CRC but with controversial results. This prompted us to investigate relative PBL telomere length in association with different histological findings throughout the continuum of colorectal carcinogenesis in order to reflect the whole spectrum of putative CRC development in a large study involving 2011 individuals. The study based on the Colorectal Cancer Study of Austria (CORSA), including 384 CRC cases as well as age- and gender-matched 544 high-risk adenomas, 537 low-risk adenoma patients and 546 colonoscopy-negative controls. Relative expression of telomeric repeats and the single copy reference gene, albumin (T/S ratio) was determined using monochrome multiplex quantitative PCR (MMQPCR). Telomeres were found to be significantly longer in CRC patients compared to control subjects (*P* = 3.61x10^-6^). Yet, no significant differences in telomere length could be detected for high-risk (*P* = 0.05956) and low-risk colorectal adenoma patients (*P* = 0.05224). In addition, results presented in this manuscript highlight the impact of various epidemiological factors on PBL telomere length and its involvement in CRC. However, further large studies also including colorectal adenomas are necessary to confirm these results.

## INTRODUCTION

Colorectal cancer (CRC) is the third most common cancer in men and the second most common cancer in women worldwide. In Austria, the incidence is in the top third within the European Union [1]. Despite improvements in early detection and treatments, CRC still remains a major cause of cancer-related death [2]. Development of CRC is a multi-step process involving several genetic and epigenetic events [3]. It is widely accepted that shortening of telomeres plays a role in the early steps of colorectal carcinogenesis by promoting chromosomal instability [4].

Telomeres are non-coding hexameric nucleotide repeats (TTAGGG)_n_ at the ends of all linear eukaryotic chromosomes [5, 6]. Along with the protein complex shelterin they form protective and highly conserved nucleoprotein structures that facilitate genomic stability and integrity [7]. Telomeric repeats can vary in size from 0.15 to 50 kilobases, a single-strand overhang at the 3’ end forms a specific T-loop structure which prevents degradation and end-to-end fusion [8]. The end replication problem of telomeres causes progressive shortening of telomere length accompanying cell division, usually triggering apoptosis, cellular senescence or immortalization in somatic cells [9, 10].

Peripheral blood leukocyte (PBL) telomere length is generally inversely correlated with age [10]. Due to various environmental influences [11, 12] and genetic differences [13, 14], telomere length in leukocytes varies remarkably between individuals among the same age. The onset as well as the susceptibility to age-related diseases including cancer is widely accepted to be affected by this inter-individual variation of PBL telomere length [15].

Several studies have examined telomere length in relation to risk of different cancer entities since first studies suggested an association of shorter telomeres with increased risk of malignancy [16]. Regarding CRC, a recently published meta-analysis of observational studies could not reveal a decisive association with PBL telomere length and cancer risk [17]. Considering the development of CRC via the putative adenoma-carcinoma sequence [18], shorter telomeres have been reported in individuals with advanced polyps suggesting that PBL telomere length could potentially be applied as a biomarker for high-risk polyps, being direct precursors of CRC [19]. Yet, the role of PBL telomere length in high-risk as well as low-risk colonic polyps is sparsely studied.

Our study based on the Colorectal Cancer Study of Austria (CORSA) biobank investigates PBL telomere length in 2011 individuals including 384 CRC, 544 high-risk polyps, 537 low-risk polyps and 546 colonoscopy-negative controls. We aimed to unveil a potential association of PBL telomere length with different histological findings throughout the continuum of colorectal carcinogenesis. The complex relationship between CRC and PBL telomere length as well as inconclusive findings in the literature prompted us to investigate the effect of various epidemiological factors on PBL telomere length and its involvement in CRC. Better understanding telomere length alterations and influencing epidemiological factors in CRC and its premalignant precursors could give valuable new insights into this complex disease.

## RESULTS

### Study population

In total, 2006 participants, consisting of 383 CRC patients, 542 high-risk adenoma patients, 535 low-risk adenoma patients and 546 colonoscopy-negative controls were eligible for statistical analysis. The majority of participants could be assigned to the age groups between 50 and 80 years. Characteristics of the study population are presented in Table 1, relative values refer to distribution within each group.

**Table 1:** Study population: distribution of cases and controls according to sex, age and smoking status

		Controls	CRC	High-risk adenoma	Low-risk adenoma	p-value (differences between status groups)
	N	546	383	542	535	0,9981
Sex	female	217 (40%)	151 (39%)	215 (40%)	214 (40%)	0,9725
Age	≤ 40	11 (2%)	8 (2%)	10 (2%)	9 (2%)	
	≤ 50	36 (7%)	29 (8%)	37 (7%)	38 (7%)	
	≤ 60	134 (25%)	86 (22%)	132 (24%)	132 (25%)	
	≤ 70	152 (28%)	111 (29%)	150 (28%)	159 (30%)	
	≤ 80	180 (33%)	120 (31%)	186 (34%)	182 (34%)	
	≤ 90	33 (6%)	29 (8%)	27 (5%)	15 (3%)	
Smoking	current	57 (10%)	54 (14%)	116 (21%)	93 (17%)	0,00001 ^***^
	former	163 (30%)	91 (24%)	174 (32%)	159 (30%)	
	never	309 (57%)	129 (34%)	233 (43%)	265 (50%)	
	missing	17 (3%)	109 (28%)	19 (4%)	18 (3%)	

### Relative telomere length

Relative telomere length was expressed as ratio between telomere signals (T) and single copy gene signals (S), yielding a relative T/S ratio. Efficiency for both amplicons ranged between 97-105 %. Intra-plate variation for telomere signals (T) was found to be ≤ 1.9% and ≤ 0.9% for single copy gene signals (S). Inter-plate variation ranged around 5% for telomeric repeats and around 2% for albumin gene. Samples with an average T/S ratio greater than 2.5 were not included in the study population, therefore 5 out of 2011 (corresponding to 0.2% of the study population) were not considered for analysis.

To evaluate the effect of histological status and co-variates (age, sex, and smoking status) on relative telomere length, several regression models were tested. For all regression analyses, the colonoscopy-negative control group, female sex and current smoking status were used as reference categories. In the best suitable multiple linear regression model, the effect of explanatory (independent) variables (histological finding, age, sex smoking status) on relative telomere length as dependent variable, and two-way interactions between age and sex and age and smoking, respectively, were considered. Further possible interactions with histological status were not significant and were therefore not included in the calculated model (Table 2). Due to missing smoking data, 163 patients were excluded from regression analysis.

**Table 2:** Best model for effect of independent variables (coefficients) and selected interactions on relative telomere length

Best Model: histological status + age, sex, smoking + interactions sex, smoking with age				
Coefficients:				
	Estimate	Std. Error	t value	*P*-value
(Intercept)	0.980710	0.021214	46.229	< 2 x 10^-16 ***^
CRC	0.079773	0.017166	4.647	3.61 x 10^-6 ***^
High-risk adenoma	0.026909	0.014274	1.885	0.05956.
Low-risk adenoma	0.027596	0.014207	1.942	0.05224.
Age	-0.012486	0.001487	-8.398	< 2 x 10^-16 ***^
Sex male	-0.051949	0.011790	-4.406	1.11 x 10^-5 ***^
Former smoker	0.015836	0.019358	0.818	0.41344
Never smoker	0.048524	0.018723	2.592	0.00963 ^**^
Age:Sex male	0.001750	0.001095	1.597	0.11033
Age:former smoker	0.003296	0.001603	2.055	0.03998 ^*^
Age:never smoker	0.003370	0.001514	2.226	0.02613 ^*^

Significance codes: 0 ‘^***^’ 0.001 ‘^**^’ 0.01 ‘^*^’ 0.05 ‘.’ 0.1 ‘ ’ 1.

Residual standard error: 0.229 on 1832 degrees of freedom.

Multiple R-squared: 0.1564, Adjusted R-squared: 0.1518.

F-statistic: 33.97 on 10 and 1832 DF, p-value: < 2.2 x 10^-16^.

The model includes main effects for histological status group, age, sex and smoking status plus two-way interactions between age and sex and age and smoking, respectively. Interactions with histological status were not significant and were therefore not included in the model.

### Telomere length and histological status

**Figure 1:**
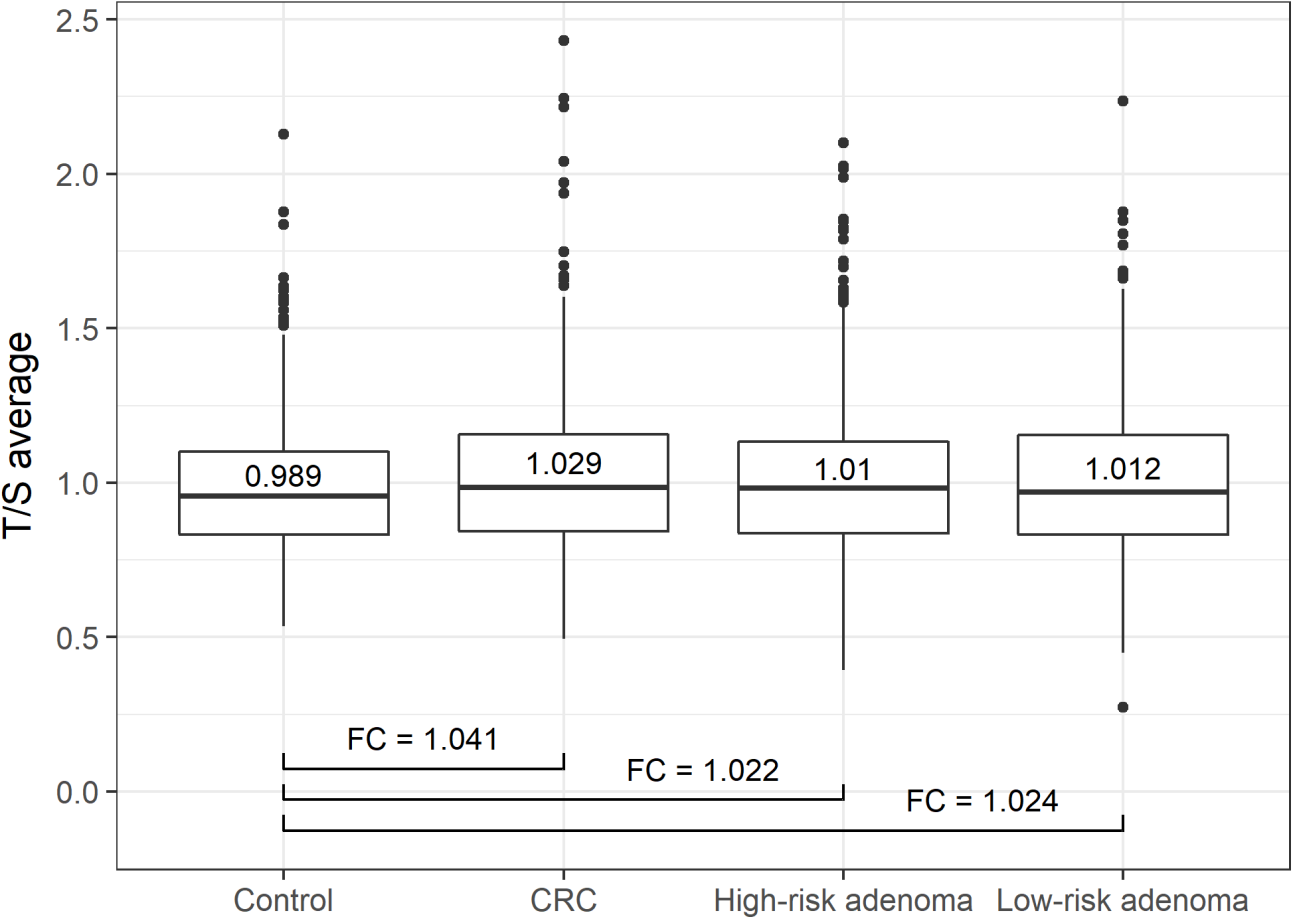
Status-wise distribution of average T/S ratios Boxplots depict the distribution of average T/S ratios of each histological status group. CRC patients presented a significantly longer telomere length than controls (*P*-value = 3.61x10^-6^). Patients with high-risk and low-risk adenoma did not significantly differ from the control group (*P*-value = 0.05956 and 0.05224, respectively). Fold changes (FCs) are calculated based on the average T/S ratio for control/CRC, control/high-risk adenoma and control/low-risk adenoma.

Patients with CRC presented a significantly longer telomere length than colonoscopy-negative controls (*P*-value = 3.61x10^-6^, Table 2). Relative telomere length ratio in high-risk and low-risk adenoma subgroups did not significantly differ from the control group (*P*-value = 0.05956 for high-risk adenoma and 0.05224 for low-risk adenoma subgroup, Table 2). In Figure 1, the status-wise distribution of average T/S ratios of each status group is depicted as boxplots. The differences of average T/S ratio compared to the control group (including confidence interval) demonstrated the significant and clearly evident variation between average T/S ratio of CRC and control group. Fold changes (FCs), calculated based on the average T/S ratio for control/CRC, control/high-risk adenoma and control/low-risk adenoma were 1.041, 1.022 and 1.024, respectively.

A general distinction between the young (≤ 64 years) and old (> 64 years) age group did not contribute to any further significant differences in average T/S ratio among patient subgroups.

### Inverse correlation between relative telomere length and age

**Figure 2:**
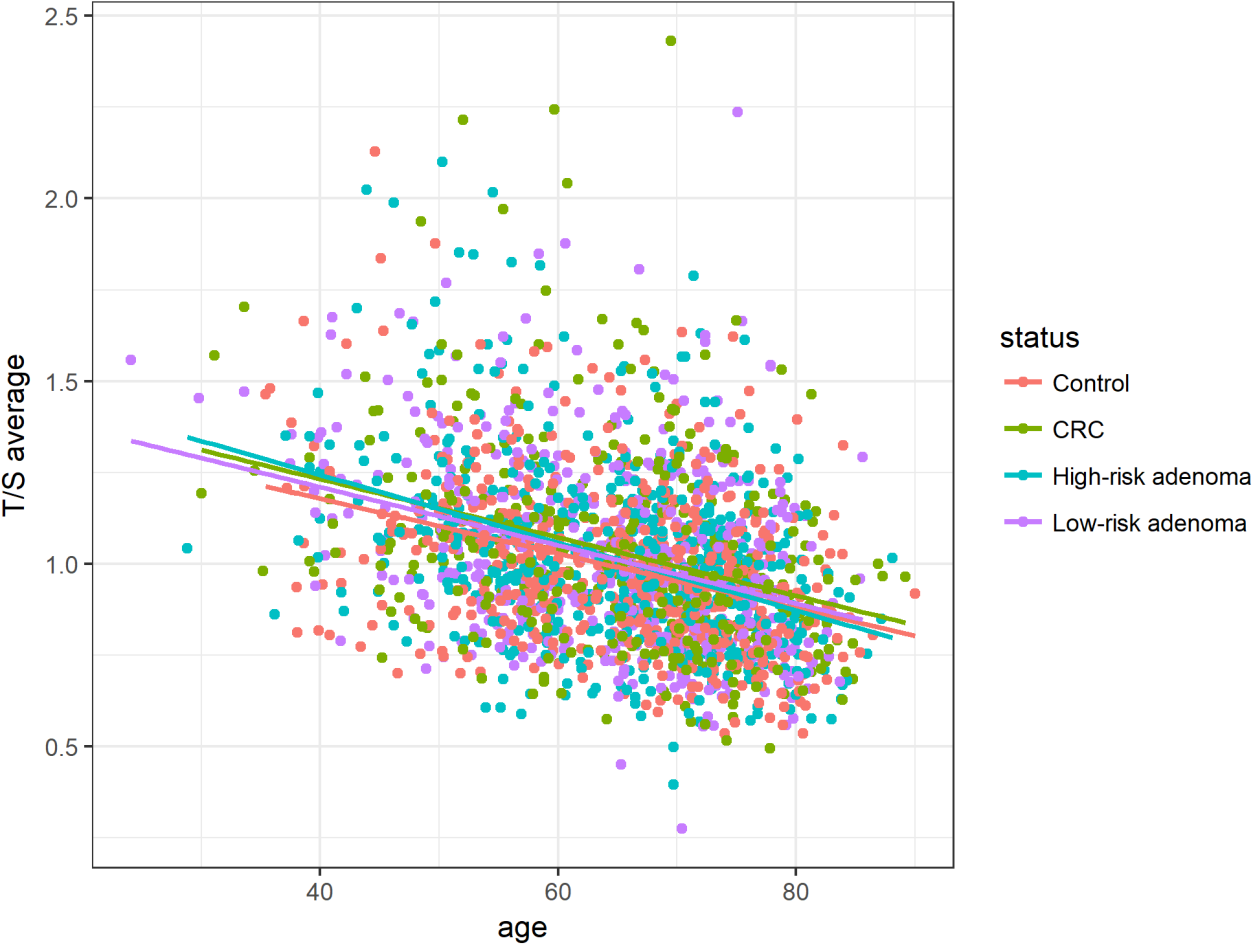
Association between age and relative telomere length for histological status groups (controls, CRC, high-risk adenoma and low-risk adenoma) Average T/S ratios were inversely associated with age among all histological status groups. Linear equations and correlation values (CV): Controls: 1.75 - 0.011 ^*^ Age (CV: -0.36), CRC: 1.88 - 0.013^*^ Age (CV: -0.32), High-risk adenoma: 1.87 - 0.013 ^*^ Age (CV: -0.39), Low-risk adenoma: 1.80 - 0.012 ^*^ Age (CV: -0.34).

Average T/S ratios were inversely associated with age among all status groups, though no interaction between cases and controls could be observed (Figure 2).

**Figure 3:**
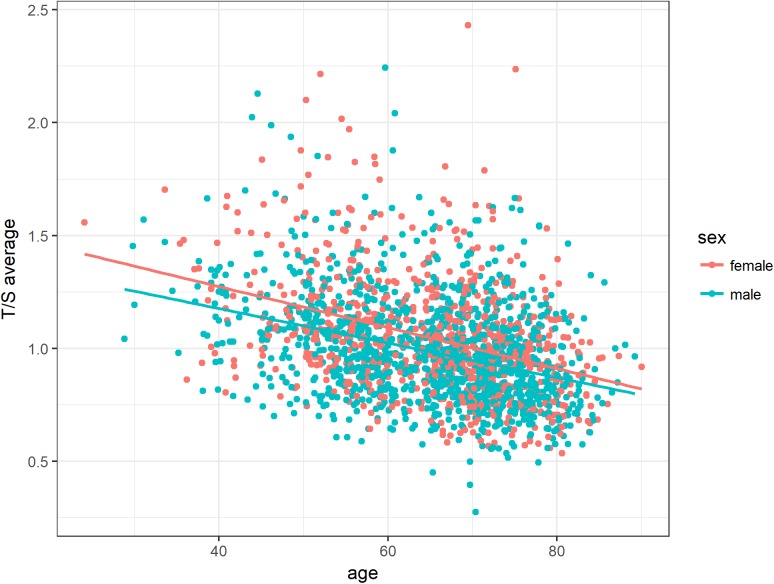
Association between age and relative telomere length for both sexes The accelerated telomere shortening with age was more pronounced in women than in men. However, this interaction was tested as not statistically significant in the regression model. Linear equations and correlation values (CV): Female: 1.64 - 0.009 ^*^ Age (-0.37), Male: 1.64 - 0.009 ^*^ Age (-0.35).

Regarding sex, the accelerated telomere shortening with age was more pronounced in female than in male subjects (Figure 3). Although female patients showed higher T/S ratios at younger age, telomere length seemed to converge to average male T/S ratios over lifetime. However, this interaction between age and sex did not reach statistical significance in the regression model (*P*-value = 0.11033, Table 2).

**Figure 4:**
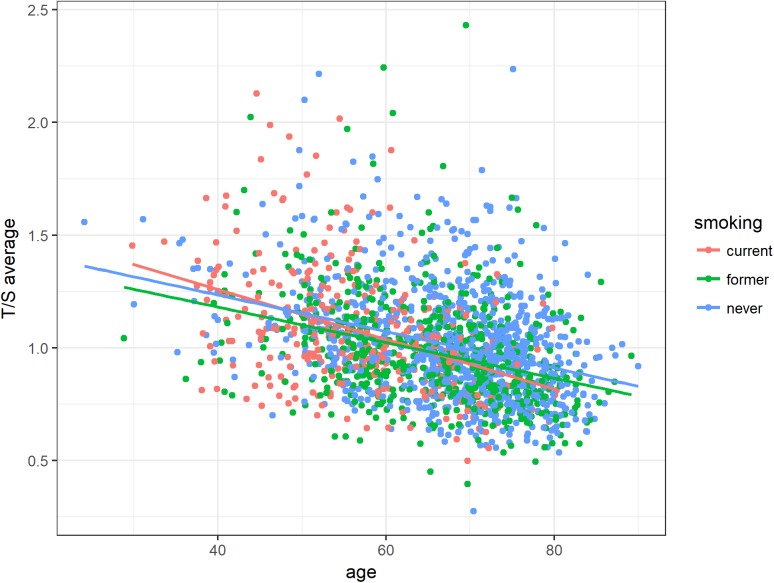
Association between age and relative telomere length according to smoking status Average T/S ratios were inversely associated with age among all smoking groups. Linear equations and correlation values (CV): current smoker: 1.70 - 0.011 ^*^ Age, former smoker: 1.50 - 0.008 ^*^ Age, never smoker: 1.56 - 0.008 ^*^ Age.

In respect of smoking status, the inverse association between telomere length and age was most remarkable for subjects that were categorized as current smokers (Figure 4).

The most significant difference in telomere shortening could be shown between patients currently smoking and patients who had never smoked (*P*-value = 0.02613, Table 2). This effect increased with age. In addition, the telomere shortening in subjects with a former history of smoking was also found significantly different from those with no smoking history (*P*-value = 0.03998, Table 2).

## DISCUSSION

Considering the continuously high prevalence and relatively high mortality of CRC globally [20], there is a strong interest in the detection of potential clinical biomarkers. Especially the investigation of PBL telomere length has been focused upon in order to identify high-risk groups for CRC and therefore spare the majority of the population colonoscopy. However, previous studies have found inconsistent associations between PBL telomere length and CRC so far.

Three prospective case-control studies could not reveal any association of telomere length with CRC [21–23]. In the retrospective SEARCH Colorectal Study [21] as well as in a retrospective case-control study within the Chinese Han population [24] shorter mean PBL telomere length was associated with CRC risk. A comparably large study comprising 598 CRC patients and 2,212 healthy controls found that younger individuals with longer PBL telomere or older individuals with shorter telomeres were associated with an increased risk for CRC [25]. Similarly, in the Shanghai Women’s Study findings indicated an elevated risk for CRC in the presence of very short and very long telomere length, analyzing telomere length in peripheral blood of 441 women with CRC and 549 matched controls in a nested case-control approach [26].

Recently, a meta-analysis by Naing et al. examined the association between PBL telomere length and CRC risk, thereby including all of the above-mentioned studies. Neither in the prospective nor in the retrospective pooled analyses, an association between PBL telomere length and CRC risk could be ascertained. As only a limited number of studies was included in this meta-analysis, the authors emphasized that there is a continued need for large well-designed studies on this topic [17].

Involving 2006 participants, our study is one of the largest investigating the association of PBL telomere length with CRC and its premalignant precursors. Here, we show that CRC patients present significantly longer PBL telomere length compared to colonoscopy-negative controls. As we have included cases as well as high- and low-risk polyps and a colonoscopy-negative control group, our study population reflects the adenoma-carcinoma sequence to capture the whole spectrum of colorectal carcinogenesis. However, no statistically significant difference for the high-risk and low-risk polyps could be demonstrated in our study.

Telomere length data on PBL throughout the continuum of colorectal carcinogenesis has not been published in the literature so far. Only a small Spanish study investigated telomere length in the adenoma-carcinoma sequence. However, telomere length of fresh tumor tissue instead of PBL was analysed from only 14 patients (6 tumor tissues, 8 polyps and normal mucosa) [27]. Regarding high-risk colorectal adenomas, one study found PBL telomere length to be shorter in patients manifesting advanced adenomas than in the colonoscopy-negative group. Nevertheless, sample sizes were considerably small, involving only 35 patients with advanced adenomas and 145 control patients. As no information on the time of blood sampling in relation to the development of advanced colorectal adenomas was provided, the validity of PBL telomere length for detection of advanced adenomas has to be considered [19].

Furthermore, in our study we also detected a significant correlation of PBL telomere length with age that is already known from vast evidence in literature [10, 11, 25, 26, 28]. Regarding sex, accelerated telomere shortening with age was generally more pronounced in women. Especially for younger female subjects a higher T/S ratio was observed compared to their male counterparts, but converged with increasing age. Age-adjusted telomere length seemed to be influenced by gender, whereby the effect of the female hormone estrogen may be accountable for longer PBL telomere length in women [29, 30].

In terms of smoking habits, the inverse association between telomere length and age was most pronounced for subjects with a currently positive smoking status. Those individuals currently smoking presented significantly stronger telomere shortening with age compared to those subjects that had never smoked. A correlation between smoking and shortened PBL telomere length has already been demonstrated [31], with telomere attrition proportional to the number of cigarettes smoked [11, 32]. Cigarette smoke triggers oxidative stress and inflammation via the generation of free radicals, which could cause telomere erosion and consequently telomere shortening [33].

Besides the large sample size, our study benefits from our homogenous population-based control group selected from the B-PREDICT screening project and therefore known to be free of polyps and CRC. An equal sample collection and processing procedure guaranteed exclusion of methodological bias between subgroups. Furthermore, we used a stable and well-established method for telomere length measurement, a monochrome multiplex quantitative PCR (MMQPCR) based assay that compares telomere repeat copy number (T) to single copy gene signals (S) (T/S ratio). Due to low intra- and interplate variation, high reproducibility and signal data consistency of our results was warranted. To exclude the fundamental confounding effect of telomere erosion following exposure to chemo- or radiotherapy [34, 35], our study only included previously untreated CRC patients to avoid modifications of PBL telomere length.

There are numerous reasons why epidemiological studies have shown controversial findings concerning a possible association between CRC risk and PBL telomere length so far. Differences are partly attributable to technical distinctions in DNA extraction methods [36], technical variations in PBL telomere length measurement methods [37], different study design e.g. retrospective versus prospective studies as well as limited study cohorts [38].

With respect to study design, a retrospective approach could have limited significance in terms of cause or consequence of telomere alterations and a possible reverse causation problem. It still remains unclear whether changes in telomere length in the blood result as a response during cancer progression or if the leukocyte telomeres itself contribute to the onset of CRC. In this regard, the time interval between sample collection and cancer diagnosis may be also responsible for differences in published data [39].

Regarding its clinical implication, telomere length of leukocyte DNA may mirror telomere length in other healthy tissues and could therefore be a potential surrogate biomarker to assess the risk for CRC [40, 41].

Here, we could report an association between longer PBL telomere length and CRC, but not for its premalignant precursors. To enhance the understanding of CRC initiation and telomere length in the blood, epidemiological telomere research has to consider the multiple telomere dynamics. Several mentioned confounders might restrain the interpretation of the complex association between PBL telomere length and CRC development. Since the inter-individual variation of telomere length is reasonably high among patients of the same age, this already indicates the difficulty of establishing a “normal range” of telomere length that could be applied as a clinical biomarker in CRC malignancy.

Yet, PBL telomere length remains a field of interest with the potential to contribute to early detection of CRC. In order to clarify the dynamics of PBL telomere length and its association with epidemiological factors in the initiation and progression of CRC, large longitudinal studies could support its evaluation and suitability as prognostic indicator of CRC.

## MATERIALS AND METHODS

### Study population

In the ongoing Colorectal Cancer Study of Austria (CORSA) more than 14,000 participants, mainly Caucasians, have been recruited since 2003 [42]. In cooperation with the province-wide screening program “Burgenland Prevention Trial of Colorectal Disease with Immunological Testing” (B-PREDICT), all inhabitants of the Austrian province Burgenland aged between 40 and 80 years are invited to participate in faecal occult blood testing (FOBT) annually. FOBT-positive tested individuals are offered a complete colonoscopy and are asked to take part in CORSA at time of colonoscopy. Further CRC cases have been recruited at the Medical University of Vienna (Department of Surgery) and in three hospitals in Vienna and the Medical University of Graz (Department of Internal Medicine).

A blood sample and questionnaires assessing anthropometric and demographic factors were obtained. Clinical data are processed in a central database following standardized documentation guidelines. All subjects gave written informed consent and the study was approved by the institutional review boards.

Participants were grouped according to their most severe histological finding: colorectal cancer (CRC), high-risk adenoma, low-risk adenoma or colonoscopy-negative controls. The high-risk adenoma group included patients with adenomatous tubular-polyps >1cm, adenomatous tubulo-villous polyps, adenomatous villous polyps, sessile serrated polyps (SSA) and traditional serrated polyps (TSA). Adenomatous tubular polyps <1cm were considered as low-risk polyps. Colonoscopy-negative controls had no polyps in their medical recording. Patients were stratified according to their age at time of blood sampling, coinciding with their most severe histological finding. Strata of five year intervals were formed ranging from the age of 40 to 90 years. Sample selection was performed on the basis of age- and gender-matched groups.

### DNA isolation

Genomic DNA of CORSA participants was routinely purified from peripheral blood using the QIAamp DNA Blood Midi Kit (Qiagen, Valencia, CA, USA) and stored in our biobank at -80°C.

### Measurement of relative telomere length (RTL)

Monochrome multiplex quantitative PCR (MMQPCR) experiments were performed to determine relative telomere length in peripheral blood leukocytes. Experiments were conducted in a high-throughput 384-well plate format (ViiA 7 instrument, Applied Biosystems, Foster City, CA, USA). An adapted version of a previously described telomere length measurement qPCR protocol was applied [43]. Two sequential Real-Time PCR programs were used to generate telomere sequences (T) as well as single copy reference gene, albumin, signals from the same well (S), corresponding to the same amount of DNA.

A DNA pool of 30 CORSA control patients (involving all age groups) served as reference to generate standard curves for telomere and albumin runs. A serial dilution (1:2) was prepared to yield seven standards. Unknown telomere fragments (T) and albumin gene (S) of samples were measured in triplicates and quantified using the respective standard curves. Relative T/S ratios were calculated and indicated relative average telomere length.

Primers Telg ACACTAAGGTTTGGGTTTGGGTTTGGGTTTGGGTTAGTGT (200 nM) and Telc TGTTAGGTATCCCTATCCCTATCCCTATCCCTATCCCTAACA (400 nm) were applied for telomere amplification; the primer pair Albugcr2 CGGCGGCGGGCGGCGCGGGCTGGGCGGCCATGCTTTTCAGCTCTGCAAGTC (200 nM) and Albdgcr2 GCCCGGCCCGCCGCGCCCGTCCCGCCGAGCATTAAGCT CTTTGGCAACGTAGGTTTC (400 nM) amplified the single copy gene albumin (Sigma-Aldrich, St. Louis, Missouri, USA). The fluorescent dye Syto 9 at a final concentration of 3 μM (Life Technologies, Carlsbad, CA, USA) and the master mix 5X HOT FIREPol Probe qPCR Mix Plus with ROX (Solis BioDyne, Tartu, Estonia) were used.

Real-time PCR conditions for telomere amplification were set as the following: 95°C/15 min, 1 cycle; 95°C/20 sec, 49°C/1 min, 2 cycles; 85°C/20 sec, 59°C/30 sec (signal acquisition), 25 cycles. For albumin gene amplification and melting curve analysis, the following thermal cycling profile was applied: 95°C/15 sec, 85°C/30 sec, 84°C/30sec (signal acquisition), 35 cycles; 95°C/15 sec, 60°C/1 min (continuous signal acquisition at 0.05°C/sec ramping); 95°C/15sec.

### qPCR quality control

PCR efficiency for both amplicons was calculated based on the slope of the standard curves, according to the equation: E= -1+ 〖10〗 ^((-1/slope)). Samples with a cycle threshold (Ct) standard deviation above 0.5 were omitted from the analysis and repeated in an independent experiment. In each run, triplicates of standard samples were included twice. This allowed monitoring of intra-plate as well as inter-plate variation.

### Statistical analysis

A multiple linear regression model was estimated with histological status, gender and smoking status as categorical predictors and age as continuous covariate. The assumptions of linear regression were assessed by diagnostic plots. Descriptive statistics of confounding factors were derived per histological status. Apart from main effects, we tested for significant two-way interactions between predictors. All tests were two-sided and p-values less than 0.05 were considered statistically significant. All statistical analyses were performed with the statistical software R version 3.33 [44].

## Abbreviations

(CRC): Colorectal cancer
(PBL): Peripheral blood leukocytes
(FC): Fold change
(CV): Correlation value
(CORSA): Colorectal Cancer Study of Austria
(MMQPCR): Monochrome multiplex quantitative PCR
(FOBT): Faecal occult blood testing
(HR): High-risk polyps
(LR): Low-risk polyps
(SSA): Sessile serrated polyps
(TSA): Traditional serrated polyps
